# Examine the association between key determinants identified by the chronic disease indicator framework and multimorbidity by rural and urban settings

**DOI:** 10.1177/26335565211028157

**Published:** 2021-06-30

**Authors:** John S. Moin, Richard H. Glazier, Kerry Kuluski, Alex Kiss, Ross E.G. Upshur

**Affiliations:** 1 University of Toronto, Institute of Health Policy Management and Evaluation (Dalla Lana School of Public Health), Toronto, ON, Canada; 2 Central Site (ICES Central), Institute for Clinical Evaluative Sciences, Toronto, ON, Canada; 3 MAP Centre for Urban Health Solutions, Li Ka Shing Knowledge Institute, St. Michael’s Hospital, Toronto, ON, Canada; 4 Department of Family and Community Medicine, University of Toronto, Toronto, ON, Canada; 5 Institute for Better Health, Trillium Health Partners, Mississauga, ON, Canada; 6 Sinai Health Systems, Toronto, ON, Canada

**Keywords:** Chronic disease, Ontario, non-communicable disease, multimorbidity

## Abstract

**Background:**

Multimorbidity, often defined as having two or more chronic conditions is a global phenomenon. This study examined the association between key determinants identified by the chronic disease indicator framework and multimorbidity by rural and urban settings. The prevalence of individual diseases was also investigated by age and sex.

**Methods:**

The Canada Community Health Survey and linked health administrative databases were used to examine the association between multimorbidity, sociodemographic, behavioral, and other risk factors in the province of Ontario. A multivariable logistic regression model was used to conduct the main analysis.

**Results:**

Analyses were stratified by age (20–64 and 65–95) and area of residence (rural and urban). A total sample of n = 174,938 residents between the ages of 20–95 were examined in the Ontario province, of which 18.2% (n = 31,896) were multimorbid with 2 chronic conditions, and 23.4% (n = 40,883) with 3+ chronic conditions. Females had a higher prevalence of 2 conditions (17.9% versus 14.6%) and 3+ conditions (19.7% vs. 15.6%) relative to males. *Out of all examined variables*, poor self-perception of health, age, Body Mass Index, and income were most significantly associated with multimorbidity. Smoking was a significant risk factor in urban settings but not rural, while drinking was significant in rural and not urban settings. Income inequality was associated with multimorbidity with greater magnitude in rural areas. Prevalence of multimorbidity and having three or more chronic conditions were highest among low-income populations.

**Conclusion:**

Interventions targeting population weight, age/sex specific disease burdens, and additional focus on stable income are encouraged.

## Background

Multimorbidity (MM) is a global phenomenon with varying patterns of disease, prevalence, and rate of increases over time.^1–3^ Consequently, the increased need for managing MM poses a major challenge to health systems and services around the world. In response, research in the field has soared globally, especially within the last two decades.^4^ In the United States, the prevalence of MM rose by 30% between 1988 and 2014.^5^ The United Kingdom projects that by 2035 the prevalence of MM could increase by 86.4%.^6^ The trends are similar in Canada, as more individuals live with MM than previously observed.^7,8^ According to recent estimates, approximately one quarter of Canadians are living with two or more chronic conditions.^1,9^ In the Province of Ontario, the prevalence of MM was nearly a quarter of the population in 2009 (24.3%), a 40% increase from 2003.^10^

Research has shown that persons with MM are at an increased risk for a wide array of social, health, and economic costs. MM has been associated with increased loneliness, social exclusion, decreased life-satisfaction and quality of life (QOL).^11–13^ Beyond loneliness and social exclusion, MM has been associated with poor mental health, cognitive and functional decline, increased disability, and death.^14–19^ Due to increased risk and association between MM, functional and cognitive decline, it is not surprising that there is also an increased probability for hospitalization, use of healthcare services, polypharmacy, and complexity of care.^20–24^

According to the Chronic Disease Indicator Framework, developed by the Public Health Agency of Canada^25^ and other research, a wide range of determinants are linked with MM: social and environmental determinants (e.g. income/material needs, stable housing, etc.); early life/childhood risk/protective factors (birth weight, breastfeeding, family violence, exposure to second hand smoke); behavioral risk/protective factors (physical activity, eating habits, sleep, stress, drug/alcohol use); other risk conditions, which are intermediate risk factors associated with chronic disease (overweight/obesity, elevated blood glucose, hypertension); and disease prevention practices (screening, vaccinations, contact with healthcare professional).^7,25–32^ The framework was developed for surveillance of chronic diseases and associated determinants in Canada, and to facilitate research and reporting.^25^ Study variables at the individual level were selected from 4 out of 6 core domains (identified by the framework), they are: social and environmental determinants, behavioral risk and protective factors, risk conditions, and health outcomes/status. More information on these core domains can be obtained elsewhere.^25^ Prenatal, early life and childhood factors were omitted due to data unavailability. Disease prevention practices were excluded as Ontario has the second highest per capita coverage of primary healthcare providers in the country with an estimated 90–93% coverage.^33,34^ Thus, while not all health determinants in this framework were tested, many were, to which there was both evidence-based justification and available data.

The following study aims to examine a comprehensive list of determinants associated with MM, which were identified by the Chronic Disease Indicator Framework and the literature. While similar studies have been conducted to date, they generally have not been as comprehensive. Roberts et al.^7^ conducted a similar epidemiological study at the national level, examining socioeconomic, health and behavioral determinants, using Canadian Community Health Survey (CCHS) (2011/12 cycle). However, their study identified cases of MM based on self-reported conditions and used nine chronic diseases, which may underestimate the prevalence in the population. In Ontario, previous research has examined MM in relation to key sociodemographic, lifestyle and health system factors by merging CCHS cycles from 2005 to 2011/12,^32^ however, life-stress and self-perceived health were not examined. In the current study, 6 CCHS cycles that are linked with Ontario health administrative databases were combined to examine additional determinants based on actual health utilization and not self-reported conditions (to identify MM cases). This study also incorporated 18 of the most common, costly, and burdensome diseases in the composite MM measure to provide improved prevalence estimates in the population. To date, no single study has examined the prevalence of MM in Ontario, to the extent proposed here.

The primary objective of this study was to examine the association between sociodemographic, lifestyle behavioral, and risk conditions with the prevalence of MM. Secondary objectives were to identify the prevalence of individual chronic diseases in the multimorbid population by age and sex.

## Methods

### Canadian community health survey (CCHS)

This study utilized several linked survey cycles (2000/01; 2003/04; 2005/06; 2007/08; 2009/10; 2011/12) from the CCHS, to analyze all eligible residents of Ontario between the ages of 20 and 95. The term “linked” implies that survey respondents’ real health utilization data has been linked to their survey with a unique encrypted id, referred to as IKN (ICES Key Number**)**. ICES is an independent, non-profit research institute which collects health care and demographic data for the purposes of health system evaluation and improvement. The CCHS is a cross-sectional survey which collects various information related to health status, health care utilization and health determinants from the Canadian population.^35^ The CCHS surveys Canadians aged 12 years and older but does not include those living in long-term care facilities, reserves, full-time members of the Canadian Armed Forces, or civilian residents on military bases.^7^ The survey was designed to derive estimates at the national and provincial levels.^7^ A complex multi-stage allocation and sampling strategy ensure relative equal weighting to health regions and the provinces.^7^ More details on the sampling methodology of CCHS are available elsewhere.^36^

For the analysis, CCHS data on sociodemographic characteristics (age, sex, rural or urban area of residence, marital status, and education) were used. Income quintiles were based on Statistics Canada’s area level income data. Household income ranges were based on CCHS survey respondents’ self-reported income. Smoking, drinking, and physical activity were included for behavioral risk and protective factors. Additional risk factors and conditions were Body Mass Index (BMI), and chronic life-stress. Self-perceived health was included as a measure for health status. Selection and classification of study determinants were based on current evidence in the field and the Chronic Disease Indicator Framework by the Public Health Agency of Canada.^25^

### Derived chronic conditions from ICES health administrative databases

MM was defined as having two or more (2+) and three or more (3+) chronic conditions from the list of 18 diseases (acute myocardial infarction (AMI), asthma, cancers, cardiac arrhythmia, chronic coronary syndrome, chronic obstructive pulmonary disorder (COPD), congestive heart failure (CHF), diabetes, hypertension, inflammatory bowel disease (IBD), mood and anxiety disorders, other mental illnesses (schizophrenia, delusions and other psychoses, personality disorders and substance abuse), osteoarthritis, osteoporosis, renal failure, rheumatoid arthritis and stroke (excluding transient ischemic attack)). The 2+ definition for MM serves as a general estimate, while the 3+ definition helps identify more high-needs patients.^37^ Chronic diseases were chosen for their high prevalence, social and economic burden, and clinical relevance.^10,38^

Health outcomes were obtained using 11 distinct health administrative databases at ICES. Where possible, validated algorithms were used to ascertain cases of: AMI, asthma, CHF, COPD, dementia, diabetes, hypertension, IBD and rheumatoid arthritis. Remaining conditions were defined based on the presence of any one inpatient hospital diagnostic code (DAD data) or two or more outpatient physician billing codes (OHIP data) within a 2-year period using ICD-9 and ICD-10 codes (Supplemental Table 1). All available data prior to index were used for deriving prevalence estimates of specified conditions, with exception of AMI (1 year prior lookback), cancer (2 years), mood disorders (2 years) and other mental illnesses (2 years), as these conditions are considered irregular and episodic.^38^

### Statistical analysis

Six linked cycles were combined and their sampling/bootstrap weights rescaled, following a similar analytical methodology from Statistics Canada.^39^ Combined rescaled weights can be treated as if one sample from the Ontario population which can be used to generate population estimates of rates, proportions, statistical models, and variances using combined bootstrap weights. Descriptive statistics of the sampled data and select variables are presented. The outcome measure for the prevalence of MM was treated as a binary dependant variable (i.e. multimorbid or not) with the index date at the time of interview (i.e. date the survey was conducted). The prevalence of MM was estimated for the general population, stratified by age, sex and determinant variables. To statistically assess the association between select determinants and the prevalence of MM, multivariable logistic regression models were used. Potential predictors and confounders were selected based on available variables, the literature, and the chronic disease indicator framework. Regression models were stratified by age (20–64 and 65–95), area of residence (rural and urban) and MM classifications (2+ and 3+). Unadjusted and adjusted odds ratios and their associated 95% confidence intervals were presented to measure association. Models were examined for multicollinearity using condition number and variance inflation factor. The maximum condition number was 5.7 which is smaller than 10, a threshold for detecting multicollinearity. The maximum variance inflation factor was 6.36 which is also smaller than 10, the cut point for multicollinearity. All analyses were weighted, p-values and confidence intervals calculated using 500 bootstrap weights provided by Statistics Canada. All tests were two-sided with p-values less than (p < 0.05) considered statistically significant. All analyses were replicated using the identified definitions of MM (2+ and 3+) composed of the prespecified chronic conditions. All analyses and forest plots were conducted and generated within SAS Enterprise Version 7.1.

### Ethics approval

This study was conducted in accordance with Research Ethics Board guidelines and policies at the University of Toronto. Studies conducted at ICES that fall under section 45 of Ontario’s PHIPA, are legally exempt from REB review, for purposes of evaluating and monitoring health systems. Furthermore, all such studies carried out within ICES are subject to a privacy impact assessment and approval from ICES Privacy and Legal Office prior to launch. The protocol for this study was approved by ICES and the data sufficiently deidentified and small cells supressed to protect privacy. All analyses for this study were conducted in a secure facility at ICES Central.

## Results

### Prevalence of multimorbidity

In the Province of Ontario, the prevalence of MM in females with 2 chronic conditions was 17.9% versus 14.6% in males, and for 3 or more chronic conditions 19.7% for females versus 15.6% for males. The prevalence of 2 and 3+ MM increased significantly in each age band for both sexes. Those without a high school degree had the highest prevalence of MM (2 & 3+ conditions = 58.5%), relative to individuals with post-secondary education (30.7%). Income showed similar patterns as education, especially for 3+ chronic conditions, as MM was most prevalent in the lowest income groups (31.7% vs. 11.6% for the highest income). There were some observed differences between rural and urban neighborhoods, with rural settings having slightly higher prevalence of MM (rural 2 & 3+ CCs 36.2%; urban 2 & 3+ CCs 33.7%). As for CCHS cycles, the prevalence of 2 conditions increased by 12.6% and 41.5% for 3+ conditions between the earliest and latest cycle.

With respect to individual factors and behaviors, those who were separated, widowed, or divorced had the highest rate of MM at 56.4%. Those reported being physically active had a prevalence rate of 30.3% for 2 and 3+ conditions versus 36.7% in the inactive population. According to BMI derived weight profiles, those who were obese had the highest prevalence of MM (2 & 3+ conditions) with 46.5%, compared with 35% in the overweight population, and 26.5% in the normal weight population. The prevalence of 3+ chronic conditions was highest in the obese population at 26.2%, compared to 12.3% in the normal weight population. Self-perceived health showed a steep increase for the prevalence of 3+ chronic conditions ranging from 5.8%-’excellent’ to 62.2%-’poor’. As for other study variables, the prevalence of MM were as follows: smoking (36.8% for 2 and 3+conditions, versus 30.2% for occasional/non-smokers); high life stress (2-conditions 19.9%; 3+ conditions 23.8%) versus little to no life stress (2-conditions 16.2%; 3+ conditions 17.4%); excess alcohol consumption (2 and 3+conditions: 30.2% non-drinkers vs. 34% regular/former drinkers). For more details see Table 1 below.

**Table 1.** Prevalence of multimorbidity by sociodemographic and individual factors.

	N Total	% Wgt	0 Conditions (%)	1 Conditions (%)	2 Conditions (%)	3+ Conditions (%)
**Sex**						
Male	78,611	48.74	28,086 (41.44)	21,127 (28.30)	12,802 (14.64)	16,596 (15.62)
Female	96,327	51.26	27,308 (34.08)	25,638 (28.36)	19,094 (17.90)	24,287 (19.66)
**Age, years**						
20–34	36,992	26.89	21,437 (60.31)	11,007 (28.76)	3470 (8.48)	1078 (2.45)
35–49	44,853	31.79	20,143 (45.52)	14,484 (32.55)	6634 (14.37)	3592 (7.57)
50–64	46,324	24.42	10,454 (23.63)	13,680 (30.55)	10,967 (23.11)	11,223 (22.71)
65–74	25,356	9.75	2389 (9.16)	5138 (20.24)	6388 (24.30)	11,441 (46.30)
75–95	21,413	7.15	971 (4.44)	2456 (11.43)	4437 (20.30)	13,549 (63.84)
**Education**						
Less than high school	23,180	8.51	3770 (20.20)	4607 (21.36)	4947 (20.59)	9856 (37.86)
High school completed	25,026	12.83	7432 (35.01)	6564 (27.22)	4740 (16.96)	6290 (20.81)
Some post-secondary	8975	5.5	3004 (38.29)	2329 (27.75)	1647 (16.58)	1995 (17.37)
Post-secondary completed	111,914	73.17	39,168 (39.85)	31,728 (29.44)	19,583 (15.81)	21,435 (14.90)
**Income group**						
0–$19,999	16,629	8.12	3202 (26.96)	3631 (23.52)	3375 (17.84)	6421 (31.68)
$20,000–$39,999	29,860	17.14	7077 (31.08)	6767 (24.14)	5925 (17.58)	10,091 (27.19)
$40,000–$59,999	26,418	18.28	8231 (36.03)	7015 (27.96)	4973 (16.99)	6199 (19.02)
$60,000–$79,999	37,623	31.62	15,074 (43.95)	11,169 (29.58)	6092 (14.84)	5288 (11.64)
$80,000+	25,048	24.85	9394 (39.01)	7830 (32.09)	4357 (16.29)	3467 (12.60)
**Income quintile**						
1 (lowest)	34,676	19.09	10,621 (39.01)	8713 (26.46)	6264 (15.60)	9078 (18.93)
2	35,039	19.36	10,972 (38.13)	9205 (27.17)	6436 (16.66)	8426 (18.04)
3	35,306	20.08	11,278 (37.58)	9452 (28.90)	6433 (15.95)	8143 (17.58)
4	35,307	20.45	11,383 (36.99)	9752 (29.46)	6372 (16.29)	7800 (17.25)
5 (highest)	34,151	21.03	11,008 (36.75)	9515 (29.49)	6302 (17.02)	7326 (16.74)
**Area of residence**						
Urban	13,6436	88.4	43,476 (37.86)	36,524 (28.43)	24,615 (16.11)	31,821 (17.60)
Rural	38,467	11.6	11,911 (36.29)	10,231 (27.55)	7272 (17.80)	9053 (18.36)
Marital status						
Married/common law	10,3924	66.35	34,026 (36.40)	29,088 (29.19)	19,002 (16.89)	21,808 (17.52)
Wid/sep/divorced	39,022	13.09	6337 (20.19)	8513 (23.42)	8515 (21.01)	15,657 (35.38)
Single	31,892	20.56	15,011 (52.87)	9136 (28.69)	4353 (11.47)	3392 (6.97)
**Smoker type**						
Regular/former	109,458	56.89	32,486 (34.97)	29,145 (28.21)	20,548 (17.31)	27,279 (19.51)
Occasional/non-smoker	65,219	43.11	22,866 (41.30)	17,560 (28.48)	11,297 (14.99)	13,496 (15.23)
**Drinker type**						
Regular/former	169,805	96.31	53,808 (37.52)	45,565 (28.45)	31,005 (16.41)	39,427 (17.61)
Never	4752	3.69	1511 (42.21)	1096 (25.09)	811 (13.68)	1334 (19.02)
**Physical activity**						
Active	85,567	48.94	29,704 (39.82)	24,741 (29.92)	15,615 (16.49)	15,507 (13.77)
Inactive	85,554	51.06	24,818 (36.19)	21,299 (27.11)	15,673 (16.21)	23,764 (20.48)
**Weight/BMI**						
Underweight	3345	2.51	1308 (48.02)	830 (27.36)	506 (11.83)	701 (12.79)
Normal weight	68,321	45.45	26,272 (44.71)	19,080 (28.81)	11,064 (14.21)	11,905 (12.27)
Overweight	57,774	34.78	17,669 (35.66)	15,862 (29.33)	10,931 (17.19)	13,312 (17.82)
Obese	32,356	17.27	7397 (26.28)	8137 (27.19)	6762 (20.32)	10,060 (26.20)
**Life stress**						
None/low stress	168,000	95.93	53,699 (38.08)	44,892 (28.40)	30,519 (16.16)	38,890 (17.36)
High stress	6472	4.07	1620 (29.07)	1782 (27.25)	1294 (19.89)	1776 (23.78)
**Self-perceived health**						
Excellent	34,843	22.16	17,042 (52.83)	10,508 (30.22)	4577 (11.19)	2716 (5.76)
Very good	63,510	37.13	23,656 (42.44)	18,958 (30.82)	11,392 (15.72)	9504 (11.01)
Good	49,622	28.09	12,402 (31.26)	12,883 (27.75)	10,367 (19.16)	13,970 (21.82)
Fair	19,313	9.1	1983 (13.97)	3390 (20.76)	4203 (21.59)	9737 (43.69)
Poor	7507	3.52	297 (4.77)	1007 (14.68)	1321 (18.35)	4882 (62.21)
**CCHS cycle year**						
2000–2001	28,370	14.91	10,620 (41.81)	7922 (28.33)	4777 (15.30)	5051 (14.57)
2003–2004	28,949	15.81	9869 (40.67)	7810 (27.70)	5191 (16.14)	6079 (15.49)
2005–2006	29,150	16.41	9728 (39.02)	7966 (28.77)	5153 (15.83)	6303 (16.38)
2007–2008	30,539	17.8	8970 (36.04)	8202 (28.66)	5818 (16.56)	7549 (18.74)
2009–2010	29,372	17.57	8454 (35.55)	7599 (28.29)	5492 (16.60)	7827 (19.57)
2011–2012	28,558	17.5	7753 (33.96)	7266 (28.21)	5465 (17.22)	8074 (20.61)

### Chronic disease prevalence by age and gender

In the multimorbid male population, the top five most prevalent conditions were: osteoarthritis (29.4%), hypertension (20.5%), asthma (11.1%), mood and anxiety disorders (9.1%), and diabetes (8.3%). For females, they were osteoarthritis (34.5%), hypertension (21.6%), mood and anxiety disorders (17.0%), asthma (13.9%), and cancer (8.9%). Stratified by age (20–64 years of age) and sex; females had twice the prevalence of cancer (8% vs. 4%), and mood and anxiety disorders (19% vs. 9.7%) than males. Males had nearly twice the prevalence rate of chronic coronary syndrome (4.4% vs. 2.4%) relative to females. In the older cohort (65–95 years of age); females had more than 6 times the prevalence of osteoporosis (18.9% vs. 2.9%) relative to males. Males had noticeably higher rates of chronic coronary syndrome (33.6% vs 22.7%). Additional age and sex variations in chronic disease prevalence can be found in Supplementary Table 2.

### Multimorbidity with 2+ chronic conditions and associated factors in rural/urban settings

After adjusting for all variables in the model, age and sex were strongly associated with MM, with the highest risk for 2+ chronic conditions in 50–64-year-old urban females (OR 5.56 CI: 5.11, 6.04) (Table 2). As expected, older age was strongly associated with MM in all the models. Urbanites who were single (OR 1.10 CI: 1.01, 1.19), separated, divorced, or widowed (OR 1.26 CI: 1.15, 1.38) had higher odds of MM than coupled individuals. This was not significant for rural residents. For older adults (65–95 years of age), only single rural residents had significantly lower odds of being multimorbid (OR 0.56 CI: 0.40, 0.79). Low-income was significantly associated with the prevalence of MM within urban settings (OR 1.17 CI: 1.03, 1.33), and showed a dose-response association in rural settings. This was not the case with older adults or rural settings. Having little to no education was a significant risk factor within urban dwelling younger adults (OR 1.15 CI: 1.03, 1.29) and for urban dwelling older adults (OR 1.20 CI: 1.04, 1.37) but not rural residents. Smoking was a significant risk factor for MM within both younger urban (OR 1.15 CI: 1.08, 1.22) and older (OR 1.17 CI: 1.04, 1.32) residents, but not for rural residents. Regular or former heavy drinking was significantly associated with MM in older rural adults (OR 1.70 CI: 1.20, 2.42) only. High life stress was significantly associated with MM for urban younger adults only (OR 1.36 CI: 1.19, 1.55). Being overweight or obese showed a strong does-response relationship for both overweight younger urban (OR 1.27 CI: 1.19, 1.36), and obese residents (OR 1.93 CI: 1.78, 2.08), and rural overweight (OR 1.38 CI:1.22, 1.56) and obese residents (OR 1.94 (1.71, 2.20). This association remained significant for older adults: urban overweight (OR 1.18 (1.05, 1.33), obese (OR 1.91 CI: 1.63, 2.24) and rural residents overweight (OR 1.27 CI:1.08, 1.50), obese (OR 2.06 CI: 1.63, 2.59). Self-perceived health showed a strong dose-response relationship within all models, with the highest odds for MM within older rural dwelling adults who self-rated their health as ‘poor’ (OR 15.51 CI: 8.99, 26.76). For more details see Table 2 below.

**Table 2.** Analysis of association between study variables and multimorbidity (2+ chronic conditions).

Variable	category	20–64 years				65–95 years			
		Urban		Rural		Urban		Rural	
		Adj OR (95%CI)	p value	Adj OR (95%CI)	p value	Adj OR (95%CI)	p value	Adj OR (95%CI)	p value
Gender	Female	1.56 (1.47, 1.66)	<0.0001	1.54 (1.39, 1.71)	<0.0001	1.14 (1.02, 1.28)	0.0264	1.03 (0.87, 1.22)	0.7131
	Male	1.0		1.0		1.0		1.0	
Age	35–49	2.05 (1.89, 2.22)	<0.0001	2.01 (1.73, 2.34)	<0.0001	—	—	—	—
	50–64	5.56 (5.11, 6.04)	<0.0001	5.04 (4.37, 5.81)	<0.0001	—	—	—	—
	20–34	1.0		1.0		—	—	—	—
	75–95	—	—	—	—	2.13 (1.89, 2.38)	<0.0001	2.22 (1.85, 2.63)	<0.0002
	65–74	—	—	—	—	1.0		1.0	
Marital status	Single	1.10 (1.01, 1.19)	0.0251	0.82 (0.70, 0.96)	0.0157	0.87 (0.72, 1.06)	0.1758	0.56 (0.40, 0.79)	0.0009
	Wid/Sep/Div	1.26 (1.15, 1.38)	<0.0001	1.11 (0.96, 1.28)	0.1768	1.02 (0.90, 1.17)	0.7308	1.05 (0.87, 1.26)	0.6013
	Married/Comm Law	1.0		1.0		1.0		1.0	
Income	$0–$19,999	1.17 (1.03, 1.33)	0.0165	1.38 (1.10, 1.72)	0.0049	0.94 (0.70, 1.26)	0.6899	0.86 (0.59, 1.24)	0.4143
	$20,000–$39,999	1.05 (0.95, 1.16)	0.3476	1.29 (1.08, 1.53)	0.0041	1.12 (0.91, 1.38)	0.2761	0.87 (0.63, 1.20)	0.3996
	$40,000–$59,999	1.02 (0.92, 1.12)	0.7443	1.22 (1.03, 1.46)	0.0224	1.14 (0.93, 1.41)	0.2041	0.75 (0.55, 1.04)	0.0844
	$60,000–$79,999	0.98 (0.90, 1.06)	0.6027	1.06 (0.89, 1.25)	0.5187	1.05 (0.85, 1.30)	0.6588	0.71 (0.51, 0.99)	0.0447
	$80,000+	1.0		1.0		1.0		1.0	
Incq	1 (lowest)	0.88 (0.80, 0.98)	0.0149	0.93 (0.79, 1.10)	0.3840	0.94 (0.79, 1.13)	0.5355	1.15 (0.90, 1.48)	0.2572
	2	0.97 (0.88, 1.06)	0.4572	0.95 (0.81, 1.12)	0.5512	0.84 (0.70, 0.99)	0.0416	1.09 (0.86, 1.39)	0.4570
	3	0.95 (0.87, 1.04)	0.2349	0.94 (0.79, 1.11)	0.4532	0.98 (0.83, 1.16)	0.8170	1.14 (0.90, 1.45)	0.2615
	4	0.96 (0.88, 1.05)	0.3556	1.06 (0.89, 1.26)	0.5053	0.96 (0.82, 1.13)	0.6373	1.12 (0.88, 1.42)	0.3454
	5 (Highest)	1.0		1.0		1.0		1.0	
Education	High School Completed	0.98 (0.90, 1.07)	0.6543	0.84 (0.74, 0.96)	0.0107	1.13 (0.97, 1.31)	0.1050	0.95 (0.77, 1.18)	0.6385
	Less than High School	1.15 (1.03, 1.29)	0.0121	1.04 (0.90, 1.21)	0.5938	1.20 (1.04, 1.37)	0.0112	0.95 (0.78, 1.16)	0.6072
	Some Post-Secondary	1.08 (0.96, 1.22)	0.2133	1.07 (0.86, 1.32)	0.5656	1.14 (0.88, 1.47)	0.3372	1.19 (0.84, 1.67)	0.3249
	Post-Secondary completed	1.0		1.0		1.0		1.0	
Drinker_type	Reg/For Drinker	1.08 (0.91, 1.28)	0.3936	0.98 (0.66, 1.45)	0.9131	1.23 (0.88, 1.72)	0.2164	1.70 (1.20, 2.42)	0.0030
	Never	1.0		1.0		1.0		1.0	
Smoker	Smoker/Former	1.15 (1.08, 1.22)	<0.0001	1.01 (0.91, 1.13)	0.7934	1.17 (1.04, 1.32)	0.0083	0.96 (0.82, 1.14)	0.6669
	Occ/non-smoker	1.0		1.0		1.0		1.0	
Phy_activity	Inactive	0.87 (0.82, 0.93)	<0.0001	0.96 (0.86, 1.06)	0.4104	1.14 (1.02, 1.27)	0.0233	1.17 (1.01, 1.37)	0.0420
	Active	1.0		1.0		1.0		1.0	
Stress_life	High Stress	1.36 (1.19, 1.55)	<0.0001	1.22 (0.95, 1.57)	0.1240	0.69 (0.42, 1.12)	0.1355	1.23 (0.63, 2.41)	0.5441
	None/Low Stress	1.0		1.0		1.0		1.0	
BMI	Obese	1.93 (1.78, 2.08)	<0.0001	1.94 (1.71, 2.20)	<0.0001	1.91 (1.63, 2.24)	<0.0001	2.06 (1.63, 2.59)	<0.0001
	Over Weight	1.27 (1.19, 1.36)	<0.0001	1.38 (1.22, 1.56)	<0.0001	1.18 (1.05, 1.33)	0.0058	1.27 (1.08, 1.50)	0.0045
	Under Weight	0.77 (0.62, 0.95)	0.0156	0.97 (0.65, 1.44)	0.8753	0.89 (0.60, 1.31)	0.5514	1.02 (0.63, 1.67)	0.9267
BMI_cat	Normal	1.0		1.0		1.0		1.0	
self_perc_hlt	Fair	5.33 (4.75, 5.98)	<0.0001	7.38 (6.07, 8.96)	<0.0001	6.24 (4.88, 7.97)	<0.0001	7.84 (5.92, 10.37)	<0.0001
	Good	2.62 (2.40, 2.86)	<0.0001	3.25 (2.80, 3.76)	<0.0001	3.30 (2.84, 3.84)	<0.0001	3.30 (2.65, 4.11)	<0.0001
	Poor	11.36 (9.44, 13.67)	<0.0001	11.53 (8.43, 15.76)	<0.0001	9.24 (6.04, 14.12)	<0.0001	15.51 (8.99, 26.76)	<0.0001
	Very Good	1.60 (1.47, 1.74)	<0.0001	1.85 (1.59, 2.16)	<0.0001	1.88 (1.63, 2.16)	<0.0001	1.86 (1.51, 2.28)	<0.0001
	Excellent	1.0		1.0		1.0		1.0	
Survey year		1.05 (1.03, 1.07)	<0.0001	1.08 (1.05, 1.11)	<0.0001	1.12 (1.08, 1.16)	<0.0001	1.09 (1.03, 1.16)	0.0016

### Multimorbidity with 3+ chronic conditions and associated factors in rural/urban settings

In the population having 3 or more chronic conditions (Supplementary Table 3), there was significantly higher odds for MM within the 50–64-year age band for both urban (OR 7.71 CI: 6.77, 8.77) and rural (OR 8.12 CI: 6.43, 10.25) adults, relative to the youngest age group and the previous model (2+ chronic conditions). There were higher odds for MM associated with lower income in younger urban adults (OR 1.45 CI: 1.22, 1.73) and rural residents (OR 1.97 CI: 1.46, 2.67) but not for older adults. Smoking was associated with MM for both younger (OR 1.16 CI: 1.06, 1.26) and older urban (OR 1.16 CI: 1.06, 1.28) adults. Physical inactivity was associated with MM only for older urban adults (OR 1.21 CI: 1.10, 1.32). High life stress was associated with MM for younger urban adults (OR 1.19 CI: 1.01, 1.40). Being overweight or obese was strongly associated with MM in both age groups and rural urban settings, most notably for obese urban younger adults (OR 2.11 CI: 1.90, 2.33). This was also evident for self-perceived health, with the strongest association observed within urban younger adults who self-rated their health as being ‘poor’ (OR 21.34 CI: 17.28, 26.34).

## Discussion

MM is a global phenomenon with high case prevalence in Ontario, Canada. Using data representative of the Ontario population, our analyses suggests that the overall prevalence of MM 2+ was nearly a third of the population (30.2%) for males and well over a third (37.6%) within the female population. More importantly, a greater proportion of the multimorbid population had 3+ chronic conditions versus 2 in both males and females. This trend has major implications for both the healthcare system and patients, as we know with accumulative chronic conditions adequate treatments/management, cost of care and quality of life are unfavorable.^13,20–22,24^ Females were more likely to be multimorbid and shared a higher burden of chronic diseases relative to males. MM was also highly prevalent within younger adults. While the prevalence of MM was higher in older age, a significant proportion were below 65, a trend observed in other studies.^7,10,26,40^ This also has implications, as more individuals live with more chronic disease for a longer period across the life course, with consequences for health systems, prevention, and early management strategies.^8,10,26,40,41^

We found unique and novel differences between associated determinants of MM and chronic diseases, between rural and urban settings. Findings showed that the association between low-income and MM was highest within younger rural adults (20–64 years of age) with three or more chronic conditions. The association between MM and income has been well established in other studies,^40^ however, the differences observed in this study between rural and urban settings have not. The observed difference may be due to greater poverty and lower incomes in rural areas. Furthermore, the lack of available services and supports in rural regions for low-income individuals which may be more accessible in urban areas. Education showed the reverse trend by being more of a factor in urban than rural settings. This may be due to the need for higher education and training to obtain good paying jobs in urban regions. Future studies should attempt to better explore these differences. Smoking was a significant factor for MM in both urban dwelling younger and older adults, but not for those residing in rural areas. The reason for this is not clear, and it would be difficult to interpret as temporal and special patterns of smoking are unique in Ontario. While, large metropolitan areas, such as the Greater Toronto Area and Ottawa, have the lowest smoking prevalence, smaller-sized cities have relatively higher smoking prevalence compared to rural areas, which could be showing in our analyses of urban settings.^42^ Another possible explanation may also be potential interactions between smoking and air quality in urban environments.^43^ The underlying reason cannot be inferred based on our study design. Regular alcohol consumption was associated with MM in rural older adults for 2+ conditions only. This could be a possible indication of increased alcohol abuse in rural settings, as a report by Statistics Canada,^44^ cited that Canadians residing in rural areas were more likely to report heavy drinking than those living in urban areas. Life stress may be another potentially important factor associated with MM yet understudied. For urban dwelling younger adults, high life stress was associated with 2+ MM and marginally for 3+ MM, but not in rural areas or in older adults. This also makes sense given that life stress peaks in middle age adulthood.^45,46^ Life stress has been previously associated with MM, with greater impact on low-income and middle-income individuals,^47,48^ consistent with our findings. Being overweight and obese were also major risk factors for MM, observed in all ages, settings, and number of conditions. This was expected, based on a previous study that there is both a strong and dose-response relationship between obesity and increased risk for MM over time.^49^ We found similar dose-response trends in this study with the overweight and obese populations. In addition to BMI, self-perception of health showed a strong dose-response relationship with number of conditions in all multivariable models, regardless of age or setting. The strongest association was observed in younger urban adults with 3 or more chronic conditions. Self-perception of health is an important determinant, as it relates to people’s ability to convey their sense of ‘wellbeing’ and is strongly associated with quality of life (QOL) outcomes.^50^ In the elderly, poorer rating of one’s self-health has been associated with further deficits in self-care, reduced functional capacity and dependency.^51^ A dose-response relationship was noted between self-perceived health and the number of conditions. Future studies are needed to clarify whether those with increased MM tend to report poorer self-rated health or if there are any indications that those who have poorer self-rated health are also more likely to become MM or experience a greater number of chronic conditions over time.

This study also casts light on the clustering of conditions by age and sex. Due to high prevalence of certain conditions by sex; males might benefit from preventative and management programs targeting excess weight, cardiovascular health, addiction, and mental health. For females, greater supports are possibly needed in weight management, bone/joint-health, cancer prevention and mental health. Keeping the number of chronic conditions down in the general population is imperative, with attention to specific vulnerabilities by sex.

Lastly, research findings indicated that 28% of males and females were one disease away from MM or further illness (i.e., 3+ MM) (14.6% males 17.9% females), thereby becoming more complex and costly. According to a study conducted by Thavorn et al.,^24^ between April 2009 and 2010, 67.9% of total healthcare costs in Ontario were incurred by 24.4% of the population with 2 or more chronic conditions. As healthcare expenditures continue to rise with every accumulative chronic disease, preventing or delaying further subsequent chronic illnesses within the general population and especially within low-income groups is paramount.

### Strengths and limitations

Based on the design and conduct of this study, a large sample of individuals were able to be factored in with relative ease and modest expenditure of resources, compared to primary data collection. A large survey with sampling weights may also provide more generalizable results to the wider population. Health outcomes derived from ICES linked data are likely more accurate as they are based on validated cohorts and actual utilization patterns. Moreover, algorithms designed to derive chronic diseases and other health-related estimates, often have inherent trade-offs between specificity and sensitivity for case detection. To avoid detecting a high rate of false positive cases, specificity is often higher than sensitivity.^52,53^ In practice it means that our estimates are likely underestimating the true prevalence of MM.

Additional considerations of study limitations are presented here. One, the study was cross-sectional and can only confer association between study variables. Two, independent study variables and included risk factors (i.e. smoking/drinking, physical activity, stress, etc.) were based on self-reported surveys and thus can be subject to potential biases (e.g. recall bias, response bias, social-desirability, proxy bias, and other potential inaccuracies). While potential biases may be inevitable, some steps were taken to limit their impact. Combining multiple cycles may have helped with some of those inaccuracies by increasing the sample size. Recall bias and potential inaccuracies were mitigated by using health utilization data and administrative databases to identify chronic diseases and MM. Proxy response bias was mitigated by limiting the age of respondents to over 20, who are more likely to complete the survey on their own. Three, the administrative data used depends on utilization therefore, access barriers to care can lead to underestimation of morbidities and MM. Four, recent studies have suggested that prevalence estimates for MM are generally higher when using administrative databases.^54^ Agreement between self-reported and health administrative estimates were worse with increasing number of conditions.^54^ Similar trends were observed in calculating health service utilization.^55^ While the cause of this difference is not entirely clear, it may be due to people’s inability to recall more than a few chronic conditions that are central to their daily management routines, when highly multimorbid. Therefore, data derived from administrative databases for this purpose may be superior, but further studies are needed. Some caution is warranted when comparing multiple studies using different data sources and case definitions for MM. This issue partly stems from the continual development of MM definitions, as there is no gold standard presently.^56^ Nevertheless, the analyses presented in this report is aligned with ongoing efforts to improve measurement and reporting consistency to better respond to health system and policy needs.^25,38^

Despite these limitations, our results indicate the need for a strong preventative approach to reduce the conversion rate of healthy younger adults’ from becoming multimorbid and incurring additional chronic diseases. Especially of concern were the younger populations at risk, due to low-wages, obesity, smoking, high stress, physical inactivity and poor self-perception of health. In addition, both utilization patterns and unique clustering of conditions by age and sex were identified. This can be of value in evaluating or designing interventions for prevention and management in the target populations. Particular attention must be paid to low-income younger adults residing within urban settings, as they were higher risk for MM, with a greater number of chronic diseases. This paper highlights the importance of addressing upstream determinants of health, as the Canadian healthcare system is designed as a universal safety net (albeit with many holes). While this is not an exhaustive list of determinants; we aimed to further the discourse on MM, in efforts of promoting optimal ageing and health in the general population, and to limit preventable strain on the healthcare system.

## Supplemental material

Supplemental Material, sj-docx-1-cob-10.1177_26335565211028157 - Examine the association between key determinants identified by the chronic disease indicator framework and multimorbidity by rural and urban settings Click here for additional data file. Supplemental Material, sj-docx-1-cob-10.1177_26335565211028157 for Examine the association between key determinants identified by the chronic disease indicator framework and multimorbidity by rural and urban settings by John S. Moin, Richard H. Glazier, Kerry Kuluski, Alex Kiss and Ross E.G. Upshur in Journal of Comorbidity
