# Supplementary material for: Examine the association between key determinants identified by the chronic disease indicator framework and multimorbidity by rural and urban settings
Source: J Multimorb Comorb. 2021 Jun 30;11:26335565211028157. doi: 10.1177/26335565211028157 (PMC8252380; doi:10.1177/26335565211028157)
Supplement: Supplemental Material, sj-docx-1-cob-10.1177_26335565211028157 - Examine the association between key determinants identified by the chronic disease indicator framework and multimorbidity by rural and urban settings [file sj-docx-1-cob-10.1177_26335565211028157.docx]

### Supplemental Table 1: ICD 9 & 10 codes, scale, range of values

Where applicable we use validated algorithms to ascertain cases (AMI, asthma, CHF, COPD, dementia, diabetes, hypertension, IBD and rheumatoid arthritis). All other conditions are defined based on the presence of any one inpatient hospital diagnostic code (DAD data) or two or more outpatient physician billing codes (OHIP data) within a 2 year period using relevant ICD-9 and ICD-10 codes (Section IV, below). We use all available administrative data prior to index (date of deriving prevalence estimates) to ascertain cases for each condition with the exception of AMI (1 year prior lookback), cancer (2 years), mood disorders (2 years) and other mental illnesses (2 years) as these conditions are considered episodic.

We define multimorbidity as the coexistence of (any combination of) two or more of these conditions. We also define level of multimorbidity as the number of diagnosed conditions among the 18 considered, which is commonly categorized as zero or one (no multimorbidity), two, three, four or five-plus conditions (depending on the distribution in the study population). This method of defining level of multimorbidity is preferred because there are currently no methods for creating meaningful clusters of chronic conditions, particularly where no central index condition is present.

Codes, scale or range of values

| **Condition [reference for validated algorithm]** | **ICD 9 / OHIP** | **ICD 10** | **ODB*** |
| --- | --- | --- | --- |
| Acute Myocardial Infarction (AMI) [1] | 410 | I21, I22 |  |
| Osteo- and other Arthritis: |  |  |  |
| (A) Osteoarthritis | 715 | M15-M19 |  |
| (B) Other Arthritis (includes Synovitis, Fibrositis, Connective tissue disorders, Ankylosing spondylitis, Gout Traumatic arthritis, pyogenic arthritis, Joint derangement, Dupuytren’s contracture, Other MSK disorders) | 727, 729, 710, 720, 274, 716, 711, 718, 728, 739 | M00-M03, M07, M10, M11-M14, M20-M25, M30-M36, M65-M79 |  |
| Arthritis - Rheumatoid arthritis [2] | 714 | M05-M06 |  |
| Asthma [3] | 493 | J45 |  |
| (all) Cancers | 140-239 | C00-C26, C30-C44, C45-C97 |  |
| Cardiac Arrhythmia | 427 (OHIP) / 427.3 (DAD) | I48.0, I48.1 |  |
| Congestive Heart Failure [4] | 428 | I500, I501, I509 |  |
| Chronic Obstructive Pulmonary Disease [5] | 491, 492, 496 | J41, J43, J44 |  |
| Coronary syndrome (excluding AMI) | 411-414 | I20, I22-I25 |  |
| Dementia [6] | 290, 331 (OHIP) / 046.1, 290.0, 290.1, 290.2, 290.3, 290.4, 294, 331.0, 331.1, 331.5, F331.82 (DAD) | F00, F01, F02, F03, G30 | Cholinesterase Inhibitors |
| Diabetes [7] | 250 | E08 - E13 |  |
| Hypertension [8] | 401, 402, 403, 404, 405 | I10, I11, I12, I13, I15 |  |
| IBD [9] | 555, 556 | K50, K51 |  |
| (Other) Mental Illnesses | 291, 292, 295, 297, 298, 299, 301, 302, 303, 304, 305, 306, 307, 313, 314, 315, 319 | F04, F050, F058, F059, F060, F061, F062, F063, F064, F07, F08, F10, F11, F12, F13, F14, F15, F16, F17, F18, F19, F20, F21, F22, F23, F24, F25, F26, F27, F28, F29, F340, F35, F36, F37, F430, F439, F453, F454, F458, F46, F47, F49, F50, F51, F52, F531, F538, F539, F54, F55, F56, F57, F58, F59, F60, F61, F62, F63, F64, F65, F66, F67, F681, F688, F69, F70, F71, F72, F73, F74, F75, F76, F77, F78, F79, F80, F81, F82, F83, F84, F85, F86, F87, F88, F89, F90, F91, F92, F931, F932, F933, F938, F939, F94, F95, F96, F97, F98 |  |
| Mood, anxiety, depression and other nonpsychotic disorders | 296, 300, 309, 311 | F30, F31, F32, F33, F34 (excl. F34.0), F38, F39, F40, F41, F42, F43.1, F43.2, F43.8, F44, F45.0, F45.1, F45.2, F48, F53.0, F68.0, F93.0, F99 |  |
| Osteoporosis | 733 | M81, M82 |  |
| Renal failure | 403, 404, 584, 585, 586, v451 | N17, N18, N19, T82.4, Z49.2, Z99.2 |  |
| Stroke (excluding transient ischemic attack) | 430, 431, 432, 434, 436 | I60-I64 |  |
| NOTES: | | | |
| Abbreviations: ICD = International Classification of Disease; ODB = Ontario Drug Benefit program database; OHIP = Ontario Health Insurance Plan, physician billings database; | | | |
| All available health administrative data (OHIP, DAD, ODB) prior to index is used to ascertain disease status, with the exception of AMI (1 year prior to index), Cancer (2 years), Mood Disorder (2 years) and Other Mental Illnesses (2 years) as these conditions are considered episodic. | | | |
| *ODB prescription drug records are not available for the majority of persons under the age of 65 | | | |
| AMI, Asthma, COPD, CHF, Dementia, Diabetes Hypertension, IBD and Rheumatoid Arthritis are based on validated case algorithms/ ICES cohorts (see 1-9 below, respectively). All other conditions required at least one diagnosis recorded in acute care (DAD) or two diagnoses recorded in physician billings (OHIP) within a two-year period. | | | |
| 1. Austin PC, Daly PA, Tu JV. A multicenter study of the coding accuracy of hospital discharge administrative data for patients admitted to cardiac care units in Ontario. American Heart Journal 2002;144:290–6. 2. Widdifield J, Bernatsky S, Paterson JM, Tu K, Ng R, Thorne JC, Pope JE, Bombardier C. Accuracy of Canadian health administrative databases in identifying patients with rheumatoid arthritis: a validation study using the medical records of rheumatologists. Arthritis Care Res 2013; 65(10): 1582-1591. 3. Gershon AS, Wang C, Guan J, Vasilevska-Ristovska J, Cicutto L, To T. Identifying patients with physician-diagnosed asthma in health administrative databases. Can Respir J 2009;16:183–8. 4. Schultz SE, Rothwell DM, Chen Z, Tu K. Identifying cases of congestive heart failure from administrative data: a validation study using primary care patient records. Chronic Diseases and Injuries in Canada 2013;33:160–6. 5. Gershon AS, Wang C, Guan J, Vasilevska-Ristovska J, Cicutto L, To T. Identifying Individuals with Physician Diagnosed COPD in Health Administrative Databases. Copd 2009;6:388–94. 6. Jaakkimainen RL, Bronskill SE, Tierney MC, Herrmann N, Green D, Young J, et al. Identification of Physician-Diagnosed Alzheimer’s Disease and Related Dementias in Population-Based Administrative Data: A Validation Study Using Family Physicians’ Electronic Medical Records. J Alzheimers Dis.; 2016 Aug 10;54(1):337–49 7. Hux JE, Ivis F, Flintoft V, Bica A. Diabetes in Ontario: Determination of prevalence and incidence using a validated administrative data algorithm. Diabetes Care 2002;25:512–6. 8. Tu K, Campbell NR, Chen Z-L, Cauch-Dudek KJ, McAlister FA. Accuracy of administrative databases in identifying patients with hypertension. Open Med 2007;1:e18–26. 9. Benchimol EI, Guttmann A, Mack DR, Nguyen GC, Marshall JK, Gregor JC, Wong J, Forster AJ, Manuel D. Validation of international algorithms to identify adults with inflammatory bowel disease in health administrative data from Ontario, Canada, J Clin Epidemiol. 2014; 67(8):887-96 | | | |

### Supplemental Table 2: Prevalence of individual chronic diseases by age and sex

*Conditions with total prevalence rates below <3% were dropped from the table

### Supplemental Table 3: Analysis of Association Between Study Variables and Multimorbidity (3+ Chronic Conditions)
